# Regression techniques employing feature selection to predict clinical outcomes in stroke

**DOI:** 10.1371/journal.pone.0205639

**Published:** 2018-10-19

**Authors:** Yazan Abdel Majeed, Saria S. Awadalla, James L. Patton

**Affiliations:** 1 Arms and Hands Lab, Shirley Ryan Ability Lab, Chicago, IL, United States of America; 2 Richard and Loan Hill Department of Bioengineering, College of Engineering and College of Medicine, University of Illinois at Chicago, Chicago, IL, United States of America; 3 Epidemiology and Biostatistics, School of Public Health, University of Illinois at Chicago, Chicago, IL, United States of America; University of Illinois at Urbana-Champaign, UNITED STATES

## Abstract

It is not fully clear which measurable factors can reliably predict chronic stroke patients’ recovery of motor ability. In this analysis, we investigate the impact of patient demographic characteristics, movement features, and a three-week upper-extremity intervention on the post-treatment change in two widely used clinical outcomes—the Upper Extremity portion of the Fugl-Meyer and the Wolf Motor Function Test. Models based on LASSO, which in validation tests account for 65% and 86% of the variability in Fugl-Meyer and Wolf, respectively, were used to identify the set of salient demographic and movement features. We found that age, affected limb, and several measures describing the patient’s ability to efficiently direct motions with a single burst of speed were the most consequential in predicting clinical recovery. On the other hand, the upper-extremity intervention was not a significant predictor of recovery. Beyond a simple prognostic tool, these results suggest that focusing therapy on the more important features is likely to improve recovery. Such validation-intensive methods are a novel approach to determining the relative importance of patient-specific metrics and may help guide the design of customized therapy.

## Introduction

Recovering from stroke is a highly variable process [1] that is difficult to influence or predict. There are many clinical assessments to evaluate the state of a patient and gauge his or her long-term prognosis. Some assessments are sufficiently reliable [2], though there is no widely accepted gold standard [3, 4]. In practice, a battery of clinical evaluations are conducted, each used to assess a different aspect of a patient’s condition. Common assessment areas include: (1) motor ability, such as Fugl-Meyer [5]; (2) functional performance, such as Wolf Motor Function Test [6]; and (3) self-reported motor activity, as in the case of the Motor Activity Log [7] and the Functional Independence Measure [8]. There is no consolidated outcome measure that encompasses these disparate evaluations, and there is general consensus that a combination of assessments provides the best profile of a patient [9].

The relationship between these clinical assessments and a patient’s movement features while performing a task is not fully understood, nor whether or how they are impacted by non-movement variables such as socio-demographic characteristics. Prediction of patient recovery has been an area of active research where much of the recent developments have focused on using imaging techniques to correlate changes in brain structure and perfusion patterns to clinical outcomes [10–15], or to use other neurophysiological and neuroimaging *biomarkers* to predict recovery [16, 17]. However, these approaches offer reasonable predictions of recovery only when the brain is imaged immediately following a stroke.

Recent computational work has shown promise predicting some clinical measures. However, the complex algorithms used were able to explain approximately 60% of the clinical outcome variability at best. To date, few and relatively recent studies used robots to explore the relationship between patient progress and clinical outcomes [18, 19]. There has also been recent success in using psychological priming to influence patient recovery from stroke without directly controlling for aspects of movement [20].

One difficulty associated with exploring the relationship between patient progress and clinical outcomes is the reliability of clinical outcomes [21, 22], especially in attempting to identify small changes in a patient’s condition or small differences between patients. These changes are often within the test-retest and inter-rater variability ranges for the clinical measures, making them difficult to use under these conditions. Some researchers have looked into using robots to obtain a more comprehensive set of clinical assessments [23, 24]. However, these works did not attempt to predict the most widely-accepted clinical outcomes of Upper Extremity Fugl-Meyer (UEFM) and Wolf Motor Function Test (WMFT).

Another computational challenge is the low number of patients in many of these studies. Combined with the high number of measurable assessments (features) available, few methods are available that produce reliable predictions while also pinpointing the most important features. The field of machine learning has recently offered robust tools to address these challenges. Here, we compared the top three candidate algorithms best known for their abilities to predict in this type of scenario of few observations and many features (mathematically referred to as the high p low n problems). Importantly, we then used cross-validation to assess the *certainty* of such predictive power, allowing us to gauge confidence in our results.

**Fig 1:**
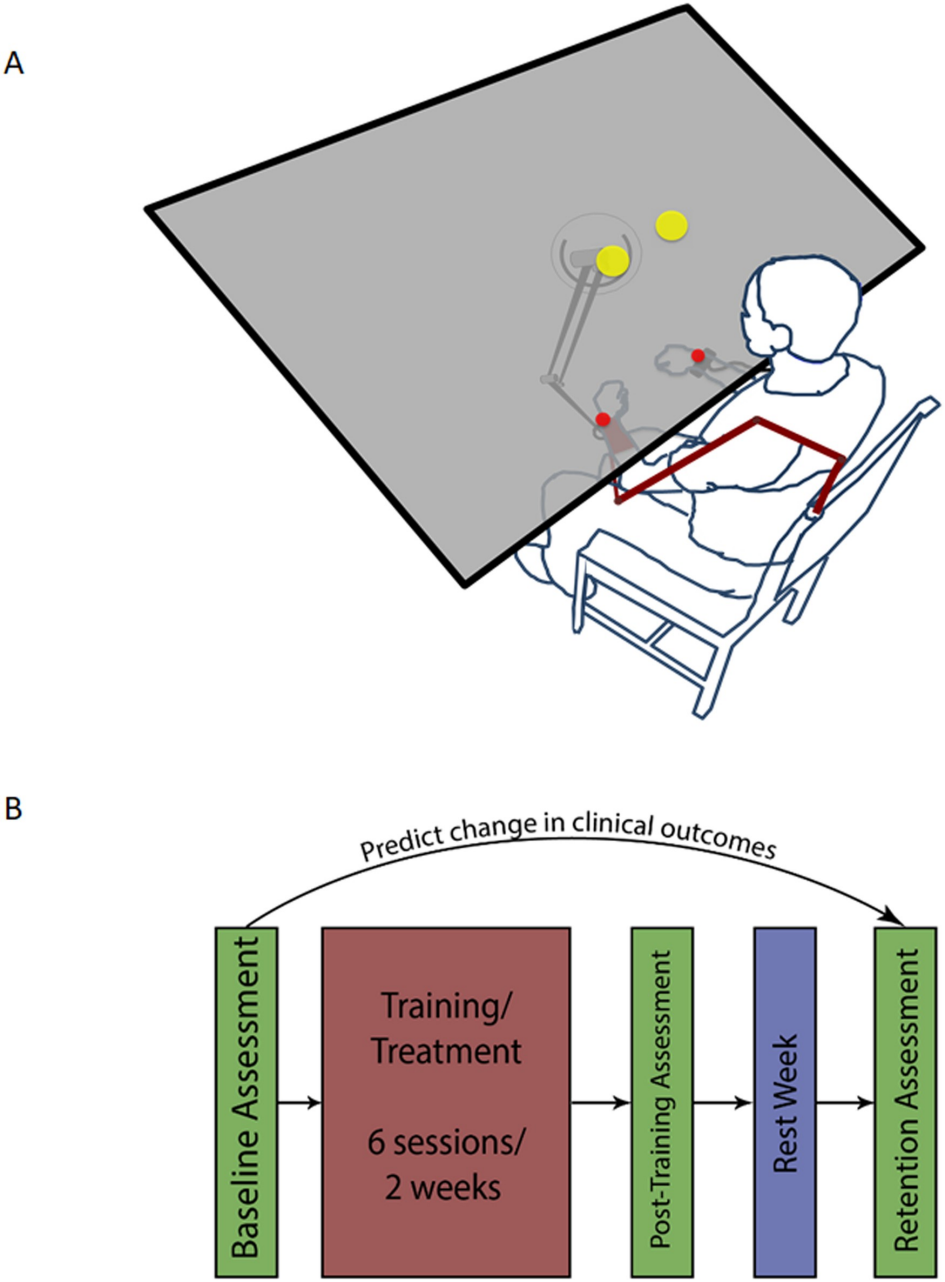
Experiment design. (A) Patients reach bimanually to two targets, pseudorandomly placed at one of four possible locations in the workspace. Patients return to a central “Home” position after every center-out reach. Patient’s wrists are represented by red spheres. Their task is to get the red spheres inside the yellow targets at the same time. (B) Patients underwent six treatment sessions over two weeks. They were evaluated prior to and immediately after training, as well as one week post-training. Our goal is to use the initial assessment, clinical, and demographic information Table 1 to predict the change in outcome measures between the baseline assessment and the final (retention) assessment.

In this study, we investigated the relationships between the changes in two typically-used clinical outcomes (Upper Extremity Fugl-Meyer, UEFM, and Wolf Motor Function Test, WMFT) following a three-week bimanual self-telerehabilitation (Fig 1A) randomized placebo-controlled intervention. We trained *N* = 26 chronic stroke survivors for a two-week period (six 1-hour training sessions, Fig 1B) and extracted variables pertaining to three domains: patient movement, clinical state and demographics (Table 1). For more details regarding the features listed in the table, please refer to the “Construction of the Feature Set” heading in our Materials and Methods section.

**Table 1:** Baseline demographic and movement features for N = 26 randomized study subjects, Collected at the Rehabilitation Institute of Chicago (Now Shirley Ryan AbilityLab) in the years 2012–2014.

Movement Features ^a^	Control Arm (*n* = 13)			Treatment Arm (*n* = 13)			Abbr.
	$\bar{mean}\pm sd$	$\bar{max}\pm sd$	$\bar{s^{2}}\pm sd$	$\bar{mean}\pm sd$	$\bar{max}\pm sd$	$\bar{s^{2}}\pm sd$ ^b^	
Reaction Time (s)	0.121 ± 0.063	0.508 ± 0.215	0.028 ± 0.023	0.196 ± 0.145	0.704 ± 0.504	0.075 ± 0.129	
Trial Time (s)	8.748 ± 2.131	9.690 ± 1.107	1.090 ± 1.266	8.630 ± 1.735	9.984 ± 0.078	2.186 ± 2.515	
Initial Direction Error (rad)	0.806 ± 0.293	2.495 ± 0.407	0.913 ± 0.336	0.915 ± 0.152	2.524 ± 0.268	1.007 ± 0.257	IDE
Pre-Movement Speed (m/s)	0.032 ± 0.021	0.120 ± 0.079	0.001 ± 0.002	0.024 ± 0.013	0.107 ± 0.074	0.001 ± 0.001	PMS
Maximum Speed (m/s)	0.264 ± 0.054	0.386 ± 0.101	0.005 ± 0.004	0.273 ± 0.069	0.407 ± 0.071	0.004 ± 0.002	
Initial Movement Ratio	0.264 ± 0.157	0.791 ± 0.153	0.080 ± 0.040	0.280 ± 0.139	0.803 ± 0.146	0.081 ± 0.031	IMR
Speed Ratio	0.571 ± 0.204	1.00 ± 0.00	0.094 ± 0.046	0.615 ± 0.167	1.00 ± 0.00	0.112 ± 0.027	
Path Length Ratio	3.448 ± 1.178	5.613 ± 2.595	1.249 ± 1.701	3.307 ± 0.999	5.913 ± 2.533	1.557 ± 1.639	PLR
Number of Speed Peaks (count)	12.81 ± 5.073	19.85 ± 7.105	16.63 ± 14.01	11.45 ± 3.220	18.38 ± 3.595	15.81 ± 9.676	NSP
Maximum Perpendicular Distance (m)	0.099 ± 0.030	0.158 ± 0.053	0.001 ± 0.001	0.089 ± 0.035	0.151 ± 0.060	0.001 ± 0.001	MPD
Percentage of Movement in the Target Direction (%)	44.4 ± 15.4	57.7 ± 14.1	0.60 ± 0.30	46.3 ± 10.5	63.5 ± 13.6	0.80 ± 0.50	PMTD
Arrest Period Ratio	0.375 ± 0.101	0.654 ± 0.138	0.022 ± 0.009	0.403 ± 0.120	0.674 ± 0.122	0.022 ± 0.011	APR
**Patient Characteristics** ^c^							
Age (yrs)		55.54 ± 12.63			55.23 ± 9.11		
Height (in)		67.62 ± 3.36			69.85 ± 4.01		
Mass (lbs)		190.08 ± 27.56			214.31 ± 47.41		
Months Since Stroke (months)		65.15 ± 70.32			64.00 ± 40.96		
Females (count)		5(38.5%)			5(38.5%)		
Left Dominant Arm (count)		3(23.1%)			2(15.4%)		
Left Affected Arm (count)		9(69.2%)			5(38.5%)		
Affected Arm = Dominant Arm (count true)		5(38.5%)			6(46.2%)		
Hemorrhagic Stroke (count)		5(38.5%)			4(30.8%)		
Cortical Lesion (count)		5(38.5%)			8(61.5%)		
Subcortical Lesion (count)		9(69.2%)			6(46.2%)		
Brainstem Lesion (count)		1(7.7%)			3(23.1%)		
Initial Fugl-Meyer Score (Fugl-Meyer Units)		38.31 ± 6.77			36.08 ± 6.86		Initial UEFM
Initial Wolf Motor Function Time Score (sec) ^d^		12.35 ± 16.40			8.59 ± 6.99		Initial WMFT
Initial Box-and-Blocks Score (number of blocks)		27.54 ± 15.15			27.00 ± 9.06		Initial BB

^a^ Features are based on 20 trials per subject

^b^ *s*^2^ denotes the variance of a feature, $\bar{s^{2}}$ represents the mean of this variance for a feature

^c^ ± notation refers to mean ± sd

^d^ WMFT is timed and therefore inversely related to ability

## Results/Discussion

Only some models were able to effectively predict clinical outcomes (WMFT and UEFM) using quadratic polynomials of the features given in Table 1, and pairwise interactions (see Methods). Next, we used only those successful models to identify and rank salient predictors of these clinical outcomes, and these rankings were consistent in 4-fold cross-validation with 100 repeats. These two steps are described in more detail in the sections below.

### Regression performance

Of the regression models we tested, we found that Least absolute shrinkage and selection operator (LASSO) [25] models performed best. We also employed *elastic nets* [26], which generalize the LASSO method, Random Forests [27], and Least Angle Regression (LARS) [28] to simultaneously conduct a sensitivity analysis of our choice of LASSO penalty and establish benchmarks for predictive ability. We relied on both the root mean square error (RMSE; S2 Fig) and the coefficient of determination (*R*^2^; S3 Fig) to quantify model performance.

LASSO’s success may be unsurprising, because it has the advantage of being able to narrow down the high-dimensional feature space to identify important features in cross-validation, and demonstrate the impact of those features on clinical outcomes. Prediction using LASSO was comparable to both the performances of LARS and a range of elastic net models with varying parameterizations S3 Fig. However, unlike elastic nets and LARS, LASSO *shrinks* the coefficient of features deemed not consequential and, thus, leads to a more parsimonious model compared to the other methods. We decided to further examine LASSO models more closely to determine the smallest subset of features that can produce the same high performance as the other methods (which tend to use many more features).

Our models predicted the change in patients’ Wolf Motor Function Test (WMFT) with better coefficient of determination (*mean* ± *sd*: 86.07% ± 5.26%) than the change in Upper Extremity Fugl Meyer (65.34% ± 17.45%). Interestingly, first-order LASSO models performed better for predicting WMFT change, while second-order models (using the base 51 features, their interactions, and quadratic terms) performed better in predicting UEFM change.

The poorer prediction performance with UEFM may be partly due to its coarse, discrete nature. That is, the continuous nature of the model prediction is more precise than the discretely reported UEFM. This may inflate the resulting RMSE values. While categorization of UEFM and the subsequent use of logistic and multinomial models may offer a remedy, there are no clear guidelines for establishing thresholds for discretizing the measures.

We found that for WMFT, first-order models mostly performed better than the more complex second-order ones S2 Fig. This is likely due to the increased likelihood of over-fitting in the second-order case, leading to poorer performance under cross-validation. The pairwise interactions and second-order terms are also likely to magnify the multicollinearity problem. These issues are well known to degrade performance of the LASSO algorithm [25, 29]. The lack of an advantage to using second-order models leads us to conclude that first-order LASSO models are sufficient for making predictions in the WMFT case. We are less confident in recommending this for the UEFM predictions because of lower predictability. An added advantage of first-order models is that they are easier to interpret and understand, and relationships between predictive features and the outcomes can be more readily translated into actionable clinical interventions.

**Fig 2:**
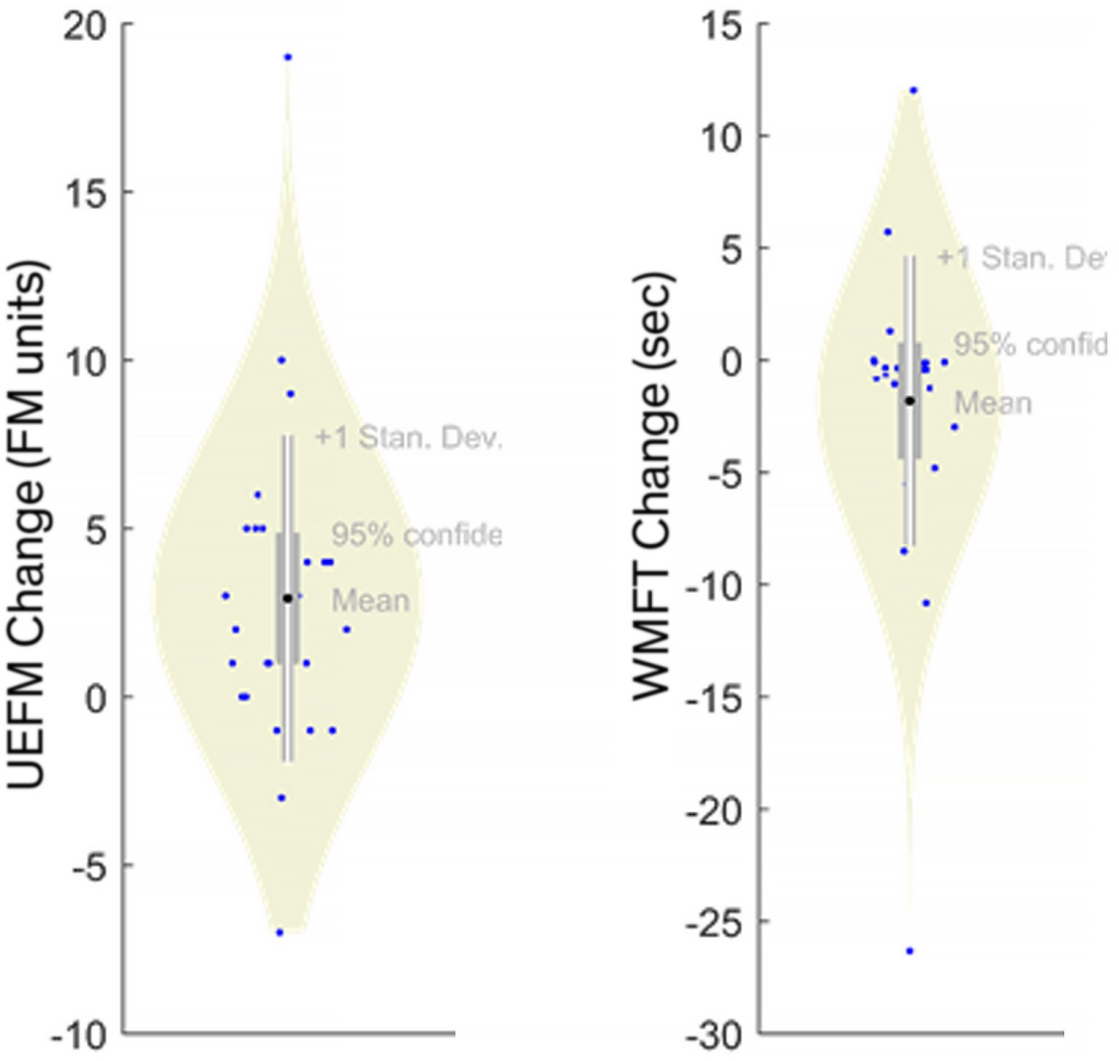
Model output distributions showing sparsity. Both UEFM and WMFT include few (sometimes one) subject(s) representing extreme values of clinical change. Clinical changes for these subjects will be difficult to predict under cross validation.

In contrast, Random Forests exhibited poor performance with very low *R*^2^ (for both first- and second-order models). *R*^2^ was < 2.24% and < 4.68% for UEFM and WMFT, respectively. These *R*^2^ values were consistent with high RMSE (≈ 5 UEFM and ≈ 6.2 WMFT). This is likely due to either collinearity or sparsity (or both). The large 51-feature input space makes this problem implicitly sparse and the sparsity of the outcomes can be observed in Fig 2, where several extreme values are represented by only a single subject. Cross validation removed points that often led to complete exclusion of some regions of the input/output spaces, worsening predictions. Collinearity by itself has little effect on a Random Forests [30, 31] because repeated random re-sampling can discriminate between collinear features; however, collinearity is likely to compound the effects of sparsity.

Another issue is that Random Forests tend to under-perform when the proportion of features that should have been selected (also called *consequential covariates*) is small ([32], section 15.3.4). Poor performance is not unreasonable if only some features are pertinent to clinical outcome, and the likelihood of randomly selecting any consequential feature at each split in a decision tree is lower. However, our lack of *a priori* knowledge of salient features motivated this analysis. Nevertheless, because of poor prediction from Random Forests, our subsequent analytic approach below focuses only on the LASSO approach.

It is important to note that UEFM and WMFT measure different aspects of movement difficulties. Not only do UEFM and WMFT have an inverse relationship, UEFM measures motor ability while WMFT measures function. Concretely, where UEFM evaluates how well patients perform fundamental actions such as bending their elbow, WMFT measures the time it takes patients to effectively perform functional tasks such as grasping and transporting an object. In the sample of patients involved in this study, while participants with higher impairment levels (low UEFM) needed more time to complete functional tasks (high WMFT) (S6 Fig).

### Feature importance

**Fig 3:**
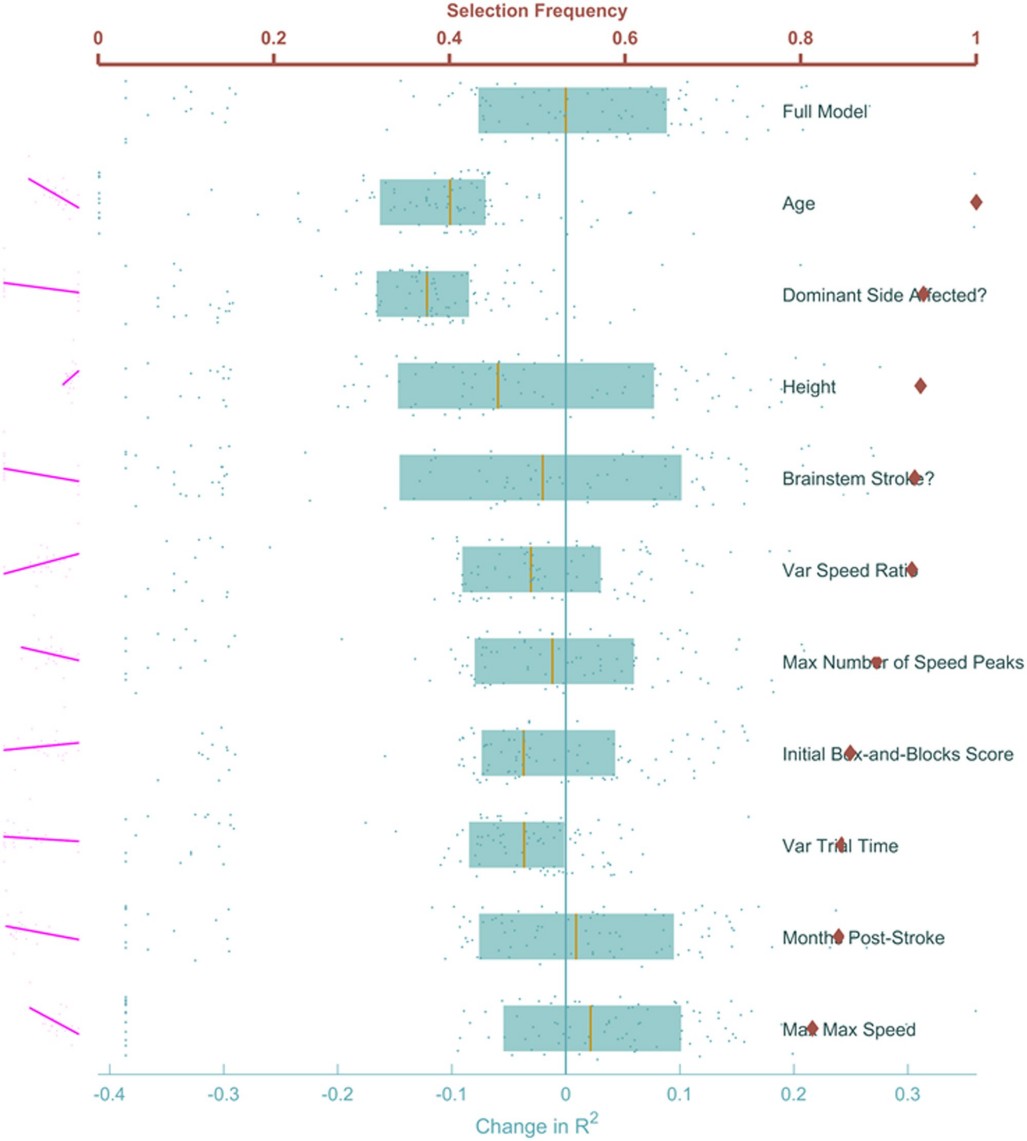
Feature ranking to predict UEFM change. Red diamonds mark the proportion of times during cross-validation where each feature was selected, with the red horizontal axis on top showing the range. The effect of removing each feature on the adjusted coefficient of determination *R*^2^ is shown in blue, each dot represents a single cross-validation run. Blue boxes show the lower quartile, median, and upper quartile of the *R*^2^ for each feature. The bottom horizontal axis measures the change in this *R*^2^ with respect to the median *R*^2^ of the full model, which is represented by the vertical blue line. The full model is shown at the top for comparison. None of the features stood out as clearly redundant or clearly essential for the model. Pairwise correlations of each feature with the outcome are shown in magenta to the left of each row.

To identify a reduced feature space we were interested in the relative importance of predictor features. We used 4-fold cross-validation enumerate how often a feature was selected (feature sufficiency), and feature omission allowed us to measure the impact of removing a feature on model prediction ability (feature necessity). The features selected most frequently by LASSO to predict UEFM change (red diamonds on Fig 3) were age (younger patients improved more), height (taller patients improved more), and affected arm (non-dominant arm improved more). Next on the list were several *movement* features related to either speed or stability: variance of speed ratio (higher variance improved more), number of speed peaks (fewer speed peaks improved more), and maximum speed (lower speeds did better). While Fig 3 shows the top 10 features, a full list of all features is shown in Supporting Information S5 Fig.

These results are consistent with previous research. It is known that cognitive performance declines with age [33]. Since height correlates with arm length [34], we posit that taller patients had an easier time reaching their virtual targets. Our results also agree with the effect of handedness on stroke recovery discussed in [35]. Higher variability leads to more comprehensive and often better learning [36]. Stroke survivors had fewer submovements as they improved [37].

Another way to gauge importance was to see how model fit was influenced by excluding a feature (blue on Fig 3). The model fit median *R*^2^ (over the 100 cross-validation runs) was most negatively impacted when either age, height or dominant side were removed, consistent with rank results above. Interestingly, removing some features resulted in changes in *R*^2^ that spanned wide ranges and even had median *improvement* (such as when maximum trial time and sex were removed), suggesting that it was better to exclude these features from consideration. With this amount of variable data, no concrete statements can be made on the importance of these features on UEFM change.

**Fig 4:**
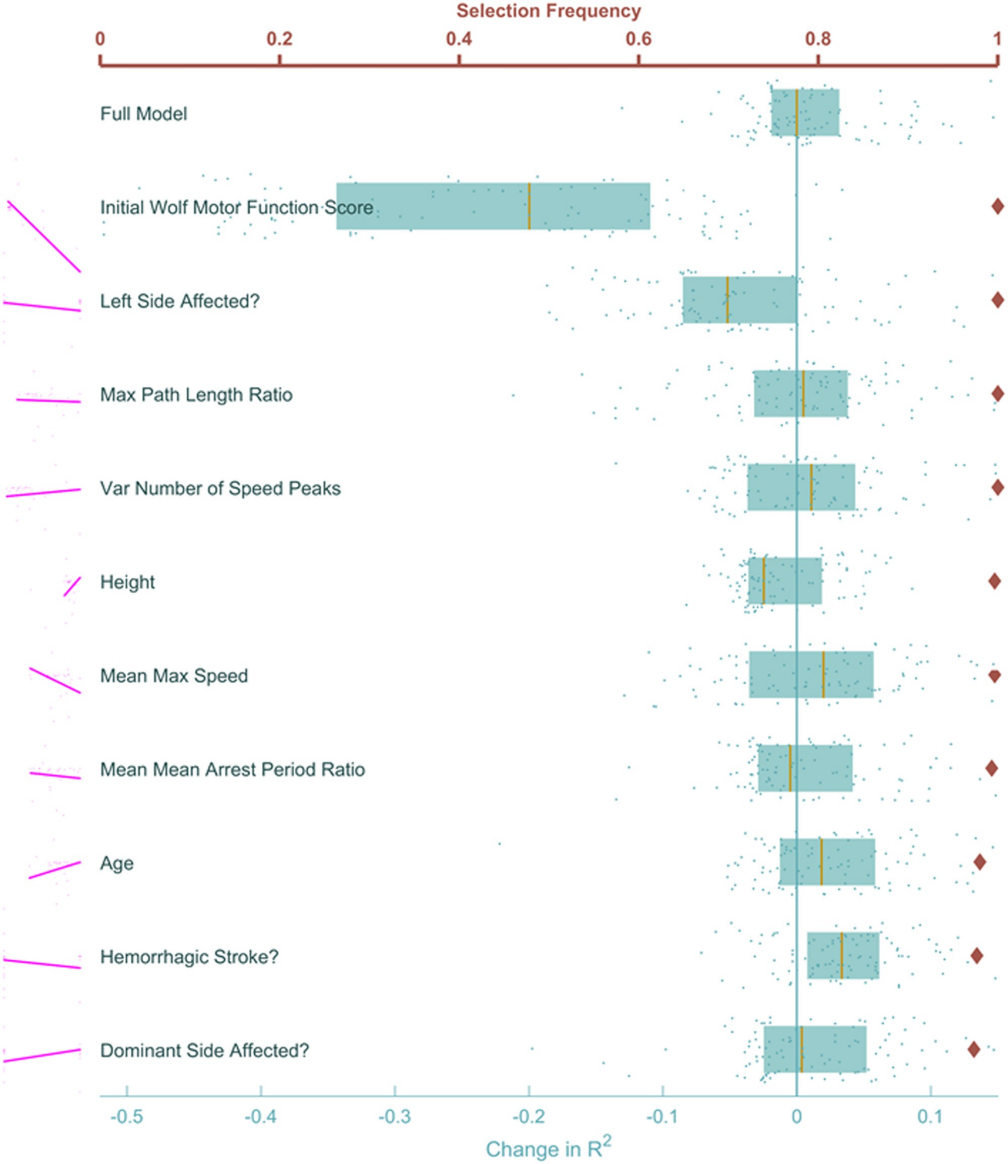
Feature ranking to predict WMFT change. Similar to Fig 3, proportion of cross-validations each feature was selected is shown in red. The blue points and boxplots show the effect of excluding each feature and rerunning the LASSO models with cross-validation. A patient’s initial WMFT score and whether their left side was affected by the stroke are the two features whose removal most negatively impacts the prediction. Conversely, removing information about the patient’s mass, stroke type and location was most helpful to the model, improving the adjusted *R*^2^. Notable among the top ten features is mean max speed, which showed a strong correlation with the outcome, indicating patients who were faster on the first day improved more on the WMFT scale.

Feature importances were more distinguishable when we inspected WMFT changes, with all of the top 10 selected by LASSO in nearly 100% of the cross-validation runs (Fig 4, Red). These were initial WMFT score (severely impaired improved more, which had the strongest correlation with WMFT change, *r* = 0.78), affected hemisphere (left side affected improved more), max path length ratio (higher ratios improved more), variance in number of speed peaks (more consistent improved more), mean max speed (this time higher speeds improved more), arrest period ratio (less time moving improved more), and age (younger improved more). It is also important to point out that mean maximum speed was next in correlation strength (*r* = 0.24).

That change in WMFT scores was best predicted by patients’ initial performance shows that the Wolf Motor test itself is a robust, consistent measure of functional recovery. The other features deemed important to the prediction indicate possible interventions to improve these WMFT changes.

These results were also supported by feature exclusions. Predictions were most negatively impacted when initial WMFT was excluded, as well as the affected hemisphere (Fig 4, blue). Because our evaluations were on the cross-validation data, the model sometimes improved when excluding a feature. This was particularly true with max and variance of the speed ratio (Fig 4). Unlike UEFM, the effects on the WMFT *R*^2^ had a smaller variance over cross-validation runs. The high variance and improvement of the mode upon their exclusion are key indicators that more data is necessary before conclusive statements regarding their importance may be made. The full list of feature ranks for predicting WMFT change is shown in the Supporting Information section S4 Fig.

In any case, features nearly always selected by LASSO were deemed essential to prediction, while features never selected were deemed ineffectual. In the central region (diamonds roughly between 0.3 and 0.7 in Figs 3 and 4) were features the LASSO model could not reliably determine were important to the model. Generally, selection of these features was contingent on which data were available to the model at the time they are considered by LASSO.

As mentioned at the beginning of this section, each of these ranking methods, though powerful, has weaknesses. When shrinking the list of features, the LASSO algorithm elects to keep the first strongly predictive features it comes across, and shrinks all features highly correlated with those to zero, as their impact is negligible given the first features are already in the model. Therefore, features chosen using this method should be treated as motifs, whereby each is interpreted not as the raw quantity it represents, but as a thematic property of a patient’s condition or ability to move that is helpful in predicting the outcome.

On the other hand, measuring the consequence of excluding an individual feature is not always accurate, because we cannot control for how other correlated features compensate for the drop in *R*^2^. One or more other factors may compensate, resulting in a small or negligible effect on *R*^2^. It is reasonable to expect that, for at least some of the features, the change in *R*^2^ reported using our method was small in spite of the importance of that feature.

Random Forests provide an estimate of feature importance that is robust against high feature correlations and not vulnerable to some of the weaknesses of LASSO for feature ranking. We were unable to use that algorithm to rank features, however, due to small number of samples. As more clinical data appears as rehabilitation science matures, such tree methods may better inform our understanding of outcome predictions and feature importance.

It is important to note that our intervention (whether the patient received Error-Augmentation treatment) was not deemed useful or important in predicting our clinical outcome scores. Surprisingly, this is in spite of showing a significant benefit to the treatment type in this randomized, controlled clinical study. If large set of features used here demonstrates that other (perhaps superfluous and uncontrolled) features are much better predictors of outcome, one questions the meaning of the classic clinical test. Truly effective interventions should appear as consistent predictors. We posit here that our validation-intensive methods can verify if detected effects are confounded by other factors.

One might be concerned about whether we would have used all the movement data across the 20 trials undertaken by each patient rather than construct our features from summary statistics, where information might be lost or hidden. However, we believe there is an advantages to using summary statistics because they can robustly resist the spurious influences of random measurement error, while also allowing for easy interpretation. Our chosen breadth of different types of summary statistics (mean, maximum, and variance) led to effective prediction models that correlate with the clinical interpretation of central tendencies, best/worst performance, and consistency.

Another concern is that only main effects and not interactions were chosen from our data. However, our fundamental goal was to identify single factors that were related to outcomes, therefore we focused our feature ranking analysis on the linear models. Interaction models not only require more data, they also are more difficult to interpret in a predictive model with no a priori selected primary exposure variable.

What is most important is the implications these results might have for patients. Younger, taller, non-dominant-affected arm individuals were more likely to improve their abilities (UEFM). Younger, more severely impaired, left arm affected individuals were more likely to improve their function (WMFT). What is also important is what these results might suggest for altering treatment strategies. While fewer speed peaks and lower maximum speeds were loosely related to ability as measured by UEFM gains, UEFM models were less successful and therefore not as reliable as the WMFT models. For the WMFT predictions, the set of highly ranked features included movement *speed*. Speed also had a strong correlation with outcome and suggests that interventions focusing on speed might improve prognosis. It remains to be seen whether a therapy that encourages faster movements might lead to better functional recovery.

## Conclusion

Changes in motor ability (UEFM) and motor function (WMFT) can both be predicted by our models. Change in WMFT is easier to predict since it is a continuous measure. Both changes in UEFM and WMFT can be linked to specific movement features as well as patient demographics and clinical characteristics. Our validation approach also allowed us to measure the certainty of our findings. Since we are unable to affect demographics or clinical characteristics, features that we can influence during rehabilitation are the most critical. This work suggests that speed would be a good first target for further study.

## Materials and methods

### Ethics statement

This work was approved by the University of Illinois at Chicago’s Institutional Review Board and Northwestern University’s Institutional Review Board. This work conforms to the Declaration of Helsinki for research involving human subjects. All participants provided written consent to participate in the study using consent procedures approved by both Institutional Review Boards.

### Patient selection & initial evaluation

We enrolled twenty-six chronic hemiparetic stroke survivors in our study. Participants had mild to moderate impairment, determined by their intake Fugl-Meyer scores (range 25-49) and were selected according to the criteria outlined in [38, 39]. Patients’ stereoscopic vision was tested using the Stereo Fly Test. Their reaching abilities were then evaluated before starting the study under similar conditions. Patients were instructed to reach with both arms in parallel, without crossing the midline, to two targets in a three-dimensional virtual reality environment (Fig 1A). Each subject underwent baseline and post-intervention evaluations consisting of a battery of clinical assessments performed by a therapist, followed by 20 bimanual reaches (trials) in the virtual reality environment, each to one of four target locations chosen pseudorandomly.

### Intervention

Patients were block-randomized controlling for age and impairment as closely as possible. Both patient groups trained for two weeks using a Phantom^®^ 3.0 robot arm. The control group received no intervention and used a passive robot arm, while the treatment group experienced disturbance to their paretic arm, in the form of visual and haptic Error Augmentation (EA) [38, 40]. All patients were evaluated again immediately after the end of training, with final evaluation taking place one week later to assess longer-term recovery effects. Our main outcome measures were changes in the patients’ clinical scores, as evaluated by a therapist, between the first evaluation (prior to beginning the study) and the final evaluation three weeks later. Specifically, our clinical outcomes were the patients’ Upper Extremity Fugl-Meyer (UEFM), which measures motor ability, and the Wolf Motor Function Test (WMFT), which measures completion time for functional tasks. This protocol is summarized in Fig 1B.

### Construction of the feature set

We gathered a total of 51 features from two sources, either demographic/physiological characteristics, and descriptive statistics of movement (Table 1). Demographic and physiological features were denoted *Z*_*il*_, *i* = 1, …, *N*, *l* = 1, …, *q*, where *q* is the number of variables, were noted at baseline. A battery of *p* measured movements were observed in *T* = 20 trials for each *i*-th subject. These measurements, *X*_*ijk*_, *j* = 1, …, *p*, *k* = 1, …, *T*, in *T* = 20, were used to compute baseline summary features across trials for each study participant: The mean (${\bar{X}}_{ij\cdot }$), maximum (*M*_*ij*⋅_), and variance(*V*_*ij*⋅_) movement features. A descriptive summary of demographic and moment variables is provided in Table 1.

Features were primarily based on common metrics or were reported in previous research [18, 41, 42]; a few of the features were newly explored in this work. These included: *(a)* performance-related measures evaluating error, speed, and reaction time, *(b)* descriptive features such as hand path length and trial time, and *(c)* patients’ demographic and clinical characteristics such as height, weight, stroke location, affected side, and initial clinical scores. Ultimately, our first-order feature set contained 51 features, and used summaries of the movement features across the 20 evaluation reaches (mean, maximum, and variance of each feature).

Most of the features we used (as detailed in Table 1) are fairly straightforward. A few are, however, somewhat ambiguous. For trial *k*, we defined speed ratio as the speed of the first launch divided by the maximum speed, while path length ratio is the distance traveled by the subject’s arm divided by the straight line distance from the home position to the target. We defined reaction time as the time between the appearance of the target and the subject crossing our pre-defined threshold of 0.06*ms*^−1^. Mean Arrest Period Ratio (MAPR) is the time the subject spent below 10% of their maximum speed for that trial divided by the total trial time. Finally, Percentage of Movement in Target Direction (PMTD) is defined as the proportion of the distance traveled during a trial in the effective direction to the target, defined formally as the sum of the projections of the distance traveled between two time samples onto the straight line path to the target, divided by the total hand path length.

### Predictive models

Movement features and patient characteristics were used to predict change in clinical outcomes: Upper Extremity Fugl-Meyer (UEFM) and Wolf Motor Function Test (WMFT). Since the number of possible predictive features is larger than the number of observations (patients), the most likely models to succeed used algorithms that shrink the number of features to avoid overfitting. These models included Elastic Net [26]. Elastic Net employs penalized linear regression with a parameter (0 ≤ *α* ≤ 1) that balances *l*_1_ and *l*_2_ norm penalties (more on that below). On one end (*α* = 0) there is ridge regression [43], purely penalizing the *l*_2_ norm of the coefficients in the model, and on the other (*α* = 1) is LASSO (Least Absolute Shrinkage and Selection Operator) [25], purely penalizing the *l*_1_ norm of the coefficients. We employed Least Angle Regression (LARS) [28]), a less greedy forward feature selection algorithm. Finally, we tried an algorithm that utilizes decision trees to make predictions (namely Random Forests [27]).

Our first-order prediction models used 51 features (many more for second-order case) to predict changes in clinical scores for 26 stroke survivors. Since this is an overdetermined problem that would be guaranteed to cause overfitting, the most likely algorithms to succeed would have to reduce the number of features used by the model. One such algorithm that we implemented was Elastic Nets, represented by the following formula:

$$\begin{array}{c} \min_{\beta_{0},\beta }\{\frac{1}{N}\sum_{i=1}^{N}{\left(y_{i}-\beta_{0}-x_{i}^{T}\beta \right)}^{2}\},\;such\;that\;\sum_{j=1}^{p*}|\beta_{j}|\leq t_{1},\sum_{j=1}^{p*}{\left|\beta_{j}\right|}^{2}\leq t_{2} \end{array}$$ (1)

where $x_{i}=[{\bar{X}}_{i1\cdot },\ldots ,{\bar{X}}_{ip\cdot },M_{i1\cdot },\ldots ,M_{ip\cdot },V_{i1\cdot },\ldots ,M_{ip\cdot }Z_{i1},\ldots ,Z_{iq}]$, p* = 3*p* + *q*, *t*_1_ and *t*_2_ are regularization terms related to the penalty, placing an upper limit on the sum of the first and second norms of predictor coefficients, and *β*_*j*_ is the coefficient of the *j*-th feature. In Lagrangian form:

$$\begin{array}{c} \min_{\beta \in R^{p*}}\{\frac{1}{N}∥y-X\beta {∥}_{2}^{2}+\alpha ∥\beta {∥}_{1}+\left(1-\alpha \right)∥\beta {∥}_{2}\}\text{s}.\text{t}.\;\alpha =\frac{\lambda_{1}}{\lambda_{1}+\lambda_{2}}, \end{array}$$ (2)

where λ_1_ *and* λ_2_ are the penalty for the sum of *l*_1_ and *l*_2_ norms of the coefficients, respectively. We tested a range of *α* values from 0 *to* 1 with increments of 0.1—where *α* = 0 corresponds to a penalty purely based on the *l*_2_ norm of the coefficients (ridge regression) and *α* = 1 corresponds to a penalty based purely on the *l*_1_ norm (LASSO regression). LASSO drives a lot of the predictor coefficients to zero and simplifies the resulting model [25] but sometimes over-regularizes. On the other hand, ridge regression keeps all coefficients in the model and drives the less useful ones close to zero, but is more difficult to interpret since it does not remove any features from the model [43]. Intermediate *α* values attempt to balance removing predictors from the model and driving their coefficients close to zero.

Least Angle Regression (LAR) adds coefficients to the model in a stepwise manner starting with the most correlated with the outcome being modeled, then adding coefficients in order of correlation with the residual from the previous step. LAR may behave in a more stable fashion than regularized regression in some cases but is also highly affected by noise [[28], discussion by Weisberg].

The chosen model in the case of regularized regression and LAR was the maximum λ where mean cross-validation error was within 1 standard error of its minimum value, as in [25].

Random Forests constructs an ensemble of decision trees, each based on a randomly chosen subset of the observations and features (therefore providing validation via random sampling, and ensuring the trees do not overfit). Each decision tree is constructed to minimize the mean square error at each split, and contributes a “vote” to the ensemble. The value predicted by the random forest for a new observation is either a majority vote (classification problems) or a mean predicted value (regression problems).

For Random Forests, we built large ensembles (50,000 regression trees) with 100 repeats of 4-fold cross validation. The large number of trees was used to ensure the algorithm has adequate usage of each feature to assess its importance, especially in the case of second-order models (> 1300 predictors).

Estimation was performed under cross-validated to avoid over-fitting and reduce the influence of outliers. Over-fitting was a concern because of the limited sample size and the eventual inclusion of second-order terms comprised of quadratic and pairwise interaction variables. We evaluated both first- and second-order models by looking at their variance explained (measured using adjusted *R*^2^) and prediction error distribution (RMSE). The process was identical for predicting both UEFM and WMFT changes. We repeated 4-fold cross-validation 100 times to obtain a range for each prediction quality metric, and ensuring we took into account different data splits.

### Ranking the features

Since LASSO regression has no built-in method for ranking model predictors and reducing the dimensionality of the input feature space needed to predict UEFM and WMFT changes, we devised two methods to unpack our LASSO models and determine the relative importance of features instrumental to clinical outcome prediction.

First, we examined the shrunk feature set resulting from each of our 100 4-fold cross-validation repeats. We used the proportion of these repeats each feature was selected as our main measure of that feature’s importance for prediction. This gave each feature a rank from 0 (always shrunk, unimportant for prediction) to 1 (never shrunk, essential for prediction).

Second, we excluded individual features and calculated the difference in prediction *R*^2^ when the feature was present and the $R_{\left(-j\right)}^{2}$ when the feature was removed. We again used 4-fold cross-validation with 100 repeats. To avoid making a distributional assumption for the change in the coefficient of variation, $\Delta_{j}=R^{2}-R_{\left(-j\right)}^{2}$, we used its median as a measure of relative importance. Features whose removal resulted in larger Δ_*j*_ were deemed more important to the model.

Random forests are a powerful method [27] for ranking features in high-dimensional data by their relative importance in predicting an outcome variable. However, we saw only limited success in their ability to identify important features in this data set. This is primarily due to their failure to effectively predict changes in clinical outcomes, likely due to the small yet highly sparse data.

## Supporting information

S1 Fig Cross-validation folds and repeats were balanced when considering individual features. There were no obvious outliers when examining each feature’s mean for each fold during cross-validation. Cross-validation means averaged to zero across all folds/repeats. This is more complex when considering second-order models, but the basic sampling in our cross-validation was balanced.
(TIF)

S2 Fig Prediction model root mean square error (RMSE). Models were successful at predicting changes in clinical outcomes, models predicting WMFT had lower errors than those predicting UEFM, Elastic Net (including LASSO and second-order Ridge) models were successful, as was LARS, while Random Forests failed. Second-order models generally did not provide an advantage over first-order models. (A) Root Mean Square Error (RMSE) results for predicting WMFT change. (B) RMSE results for predicting UEFM change.
(TIF)

S3 Fig Prediction model coefficient of determination. Models were successful at predicting changes in clinical outcomes, WMFT models performed better than UEFM, Elastic Net (including LASSO and second-order Ridge) models were successful, as was LARS, while Random Forests failed. Second-order models generally did not provide an advantage over first-order models. (A) Adjusted coefficient of determination *R*^2^ results for predicting WMFT change. (B) Adjusted coefficient of determination results for predicting UEFM change. These results are consistent with RMSE finding (S2 Fig). We saw higher mean *R*^2^ with first-order than second-order Elastic Net and LARS models, and second-order models tended to have higher variance, especially when predicting change in UEFM.
(TIF)

S4 Fig Full list of feature ranks predicting WMFT change. Proportion of cross-validations each feature was selected is shown in red. The blue points and boxplots show the effect of excluding each feature and rerunning the LASSO models with cross-validation. A patient’s initial WMFT score and whether their left side was affected by the stroke are the two features whose removal most negatively impacts the prediction. Conversely, removing information about the patient’s mass, stroke type and location was most helpful to the model, improving the adjusted *R*^2^. Notable among the top ten features is mean max speed, which showed a strong correlation with the outcome, indicating patients who were faster on the first day improved more on the WMFT scale.
(TIF)

S5 Fig Full list of feature ranks predicting UEFM change. Red diamonds mark the proportion of times during cross-validation where each feature was selected, with the red horizontal axis on top showing the range. The effect of removing each feature on the adjusted coefficient of determination *R*^2^ is shown in blue, each dot represents a single cross-validation run. Blue boxes show the lower quartile, median, and upper quartile of the *R*^2^ for each feature. The bottom horizontal axis measures the change in this *R*^2^ with respect to the median *R*^2^ of the full model, which is represented by the vertical blue line. The full model is shown at the top for comparison. None of the features stood out as clearly redundant or clearly essential for the model. Pairwise correlations of each feature with the outcome are shown in magenta to the left of each row.
(TIF)

S6 Fig UEFM and WMFT have an inverse relationship. UEFM was more sensitive to patients with relatively higher functional ability, while WMFT was more sensitive to those with lower functional ability. WMFT scores plateaued for patients showing larger UEFM changes. This relationship between UEFM and WMFT may explain our observation that slower speed predicted better recovery for UEFM while higher speeds were predictive of faster WMFT times. Changes in clinical scores were not statistically significant after the intervention.
(TIF)
