# Supplementary figures and images for: Regression techniques employing feature selection to predict clinical outcomes in stroke
Source: PLoS One. 2018 Oct 19;13(10):e0205639. doi: 10.1371/journal.pone.0205639 (PMC6195279; doi:10.1371/journal.pone.0205639)

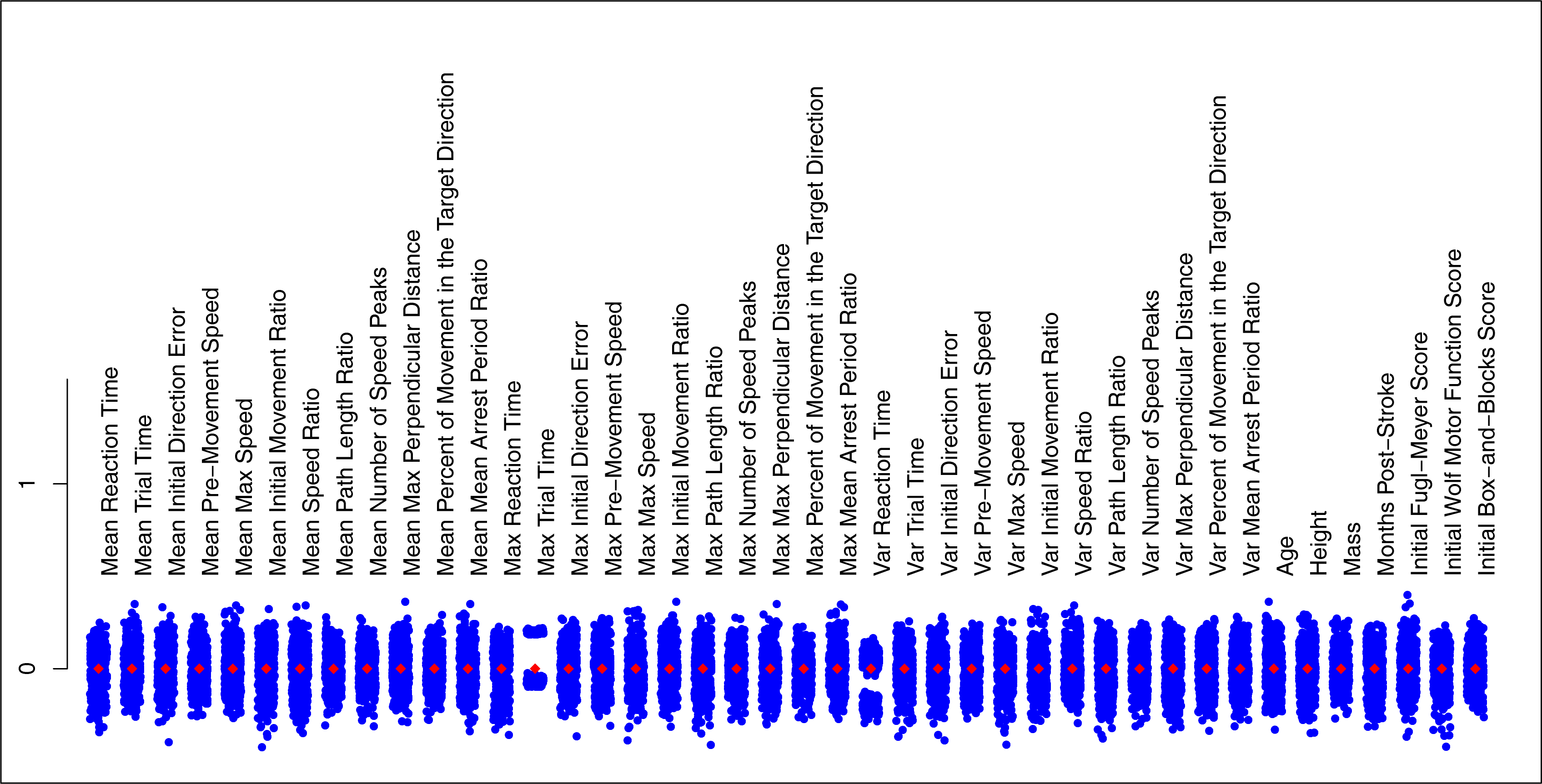

Supplement: S1 Fig — There were no obvious outliers when examining each feature’s mean for each fold during cross-validation. Cross-validation means averaged to zero across all folds/repeats. This is more complex when considering second-order models, but the basic sampling in our cross-validation was balanced. (TIF) [file pone.0205639.s001.tif]

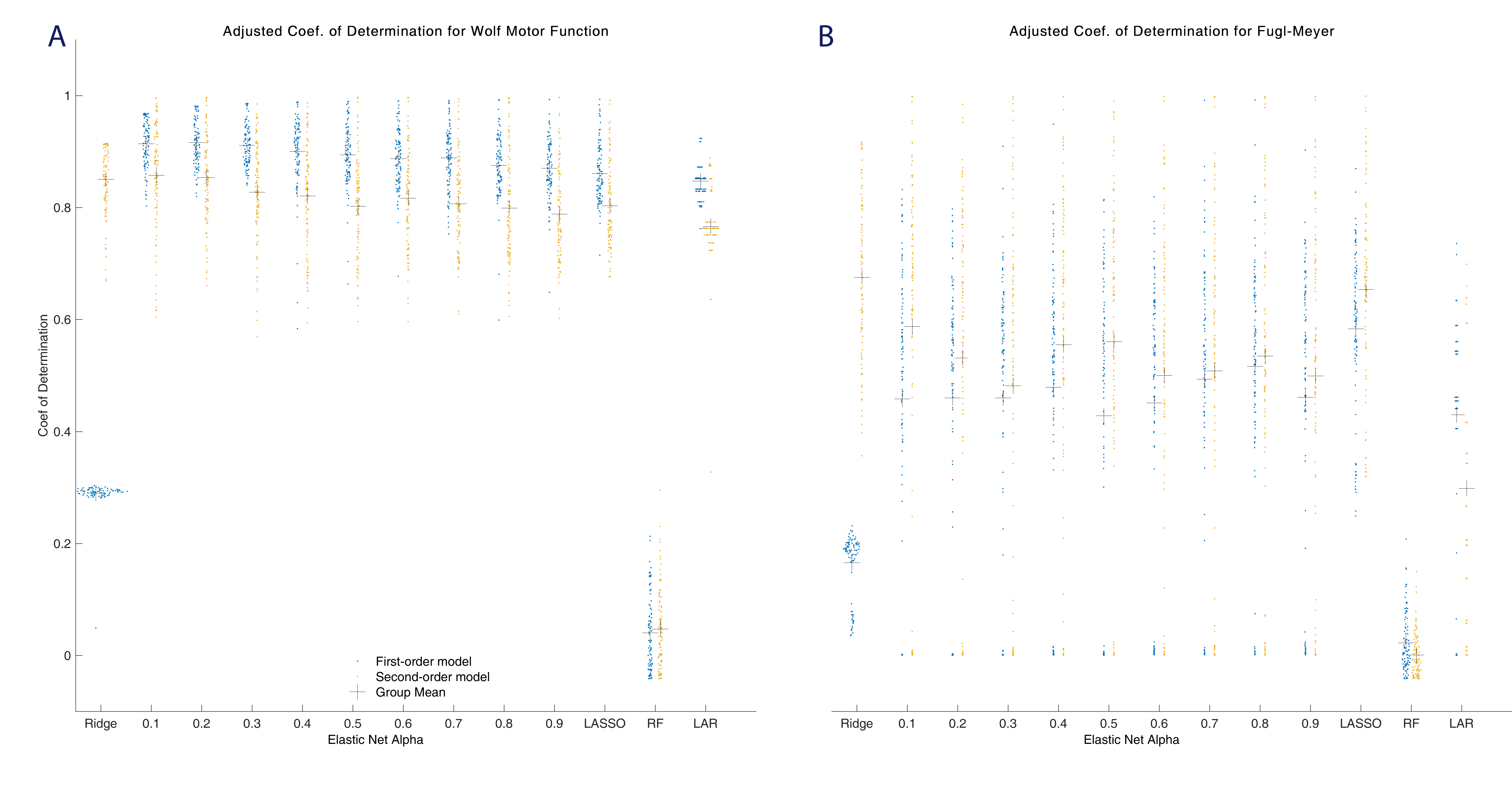

Supplement: S2 Fig — Models were successful at predicting changes in clinical outcomes, models predicting WMFT had lower errors than those predicting UEFM, Elastic Net (including LASSO and second-order Ridge) models were successful, as was LARS, while Random Forests failed. Second-order models generally did not provide an advantage over first-order models. (A) Root Mean Square Error (RMSE) results for predicting WMFT change. (B) RMSE results for predicting UEFM change. (TIF) [file pone.0205639.s002.tif]

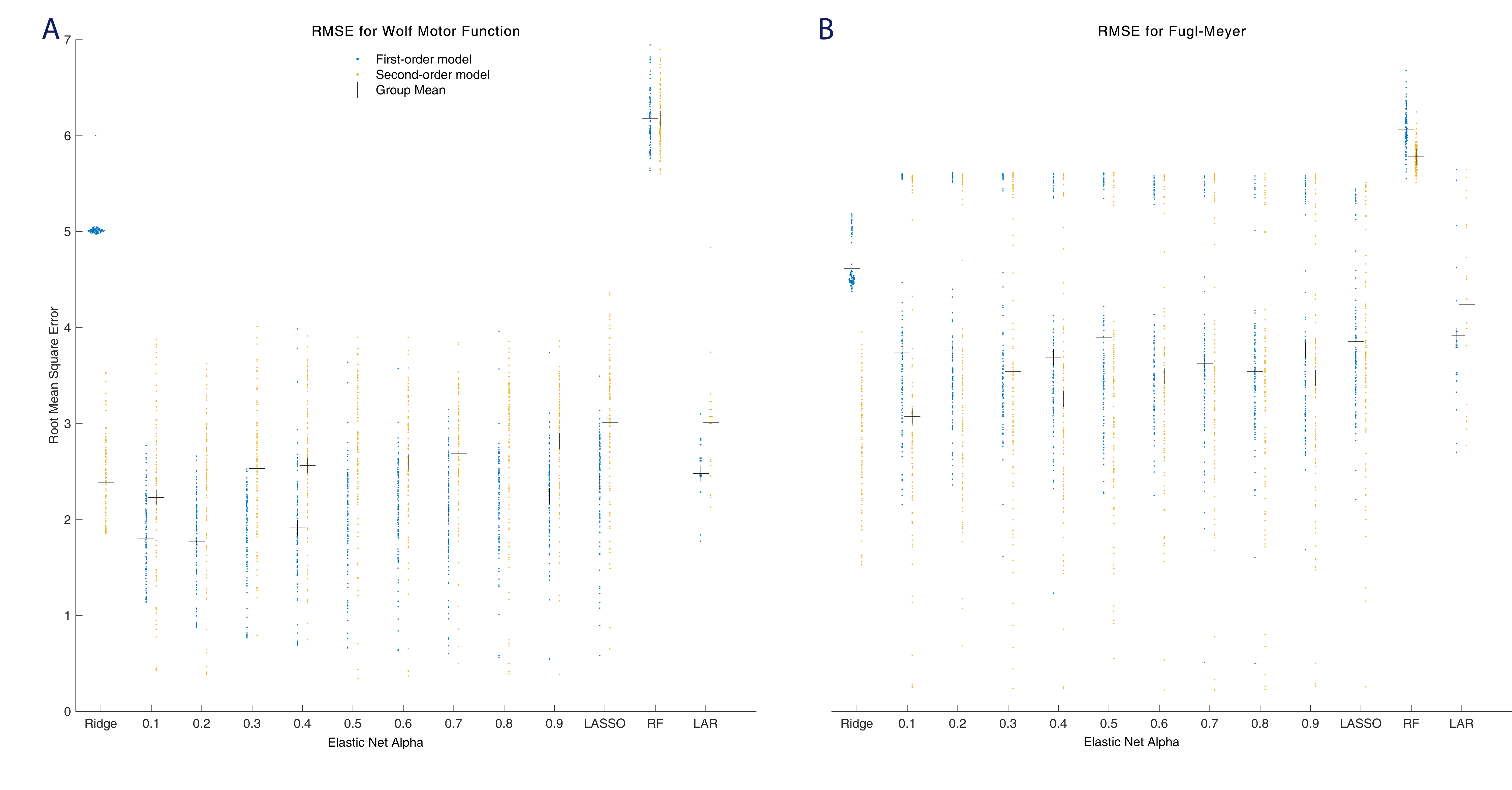

Supplement: S3 Fig — Models were successful at predicting changes in clinical outcomes, WMFT models performed better than UEFM, Elastic Net (including LASSO and second-order Ridge) models were successful, as was LARS, while Random Forests failed. Second-order models generally did not provide an advantage over first-order models. (A) Adjusted coefficient of determination R2 results for predicting WMFT change. (B) Adjusted coefficient of determination results for predicting UEFM change. These results are consistent with RMSE finding (S2 Fig). We saw higher mean R2 with first-order than second-order Elastic Net and LARS models, and second-order models tended to have higher variance, especially when predicting change in UEFM. (TIF) [file pone.0205639.s003.tif]

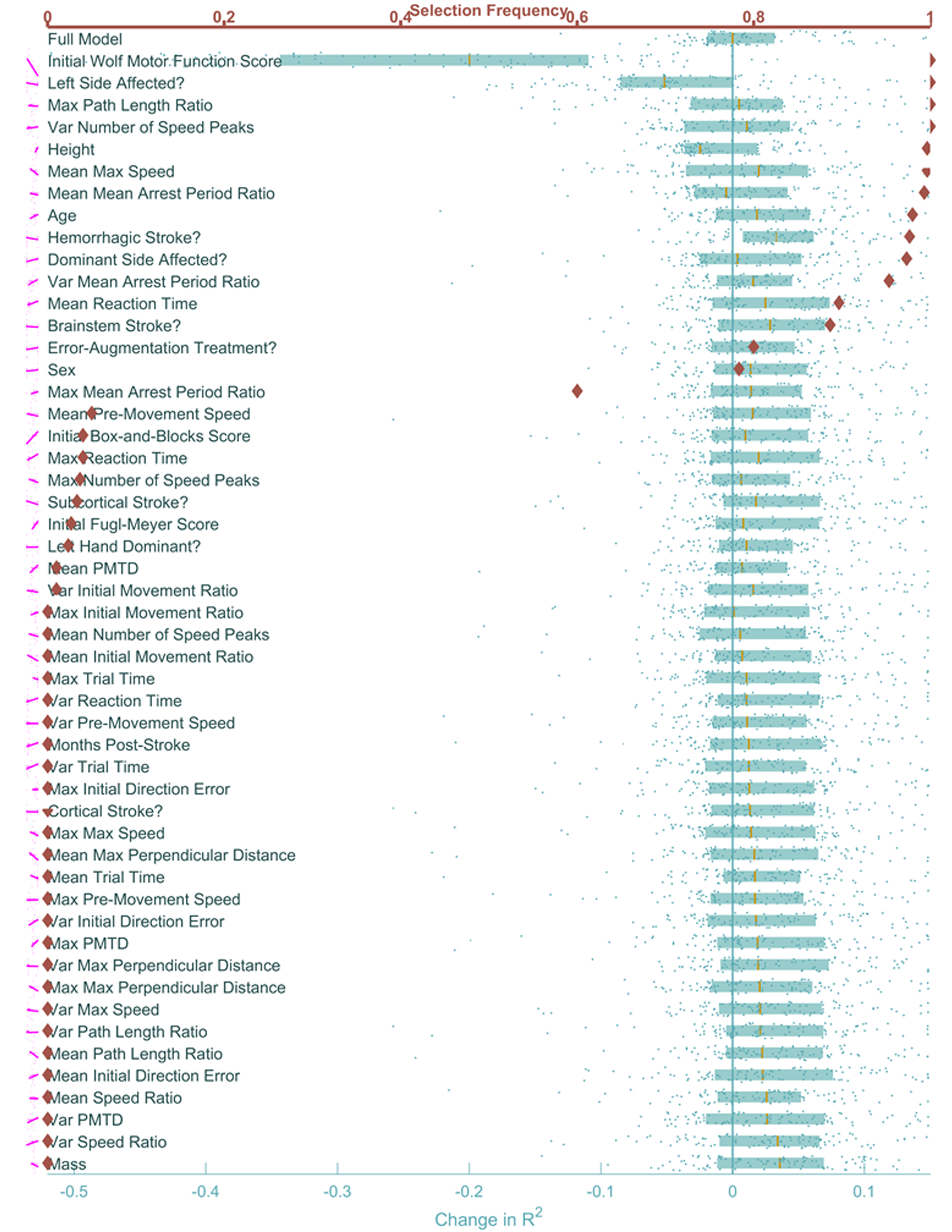

Supplement: S4 Fig — Proportion of cross-validations each feature was selected is shown in red. The blue points and boxplots show the effect of excluding each feature and rerunning the LASSO models with cross-validation. A patient’s initial WMFT score and whether their left side was affected by the stroke are the two features whose removal most negatively impacts the prediction. Conversely, removing information about the patient’s mass, stroke type and location was most helpful to the model, improving the adjusted R2. Notable among the top ten features is mean max speed, which showed a strong correlation with the outcome, indicating patients who were faster on the first day improved more on the WMFT scale. (TIF) [file pone.0205639.s004.tif]

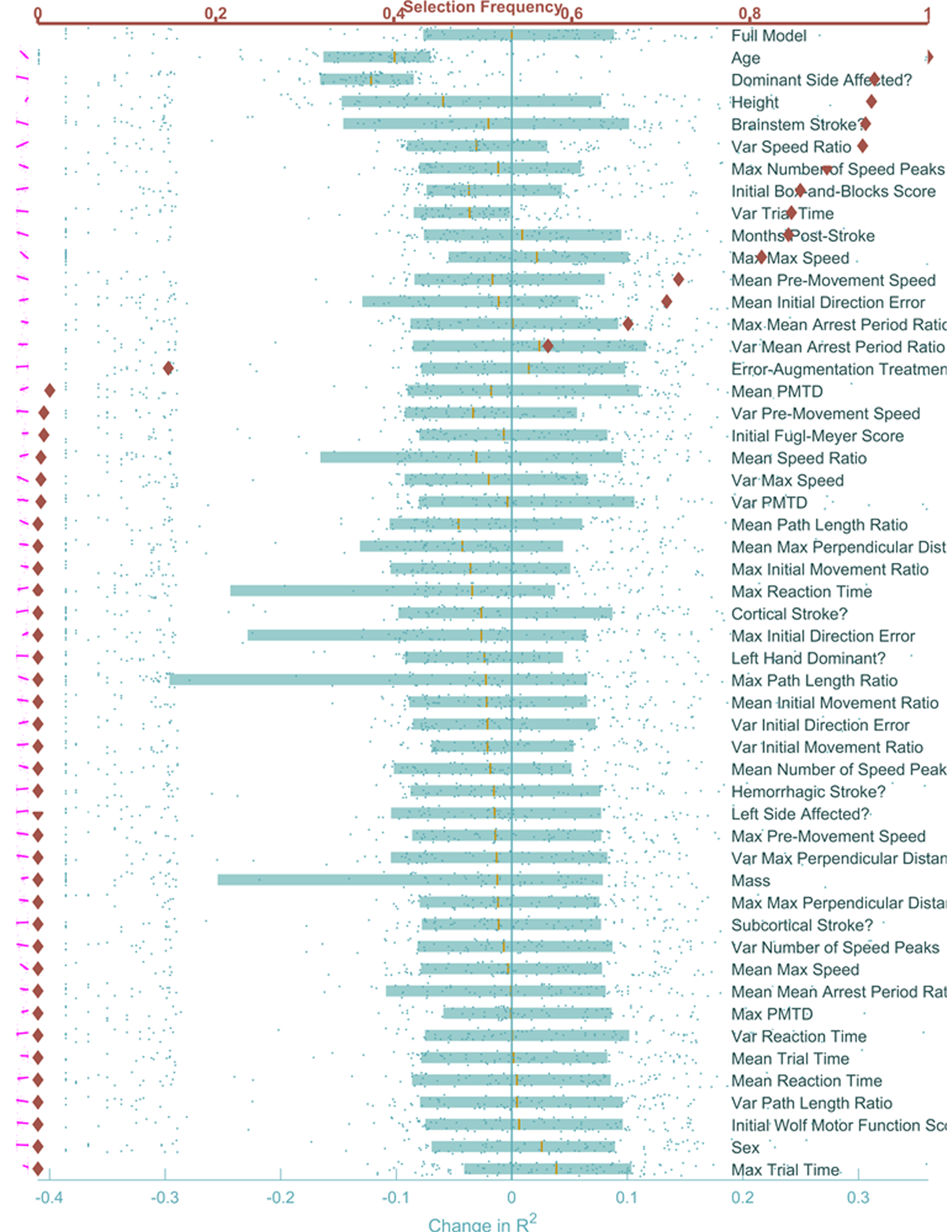

Supplement: S5 Fig — Red diamonds mark the proportion of times during cross-validation where each feature was selected, with the red horizontal axis on top showing the range. The effect of removing each feature on the adjusted coefficient of determination R2 is shown in blue, each dot represents a single cross-validation run. Blue boxes show the lower quartile, median, and upper quartile of the R2 for each feature. The bottom horizontal axis measures the change in this R2 with respect to the median R2 of the full model, which is represented by the vertical blue line. The full model is shown at the top for comparison. None of the features stood out as clearly redundant or clearly essential for the model. Pairwise correlations of each feature with the outcome are shown in magenta to the left of each row. (TIF) [file pone.0205639.s005.tif]

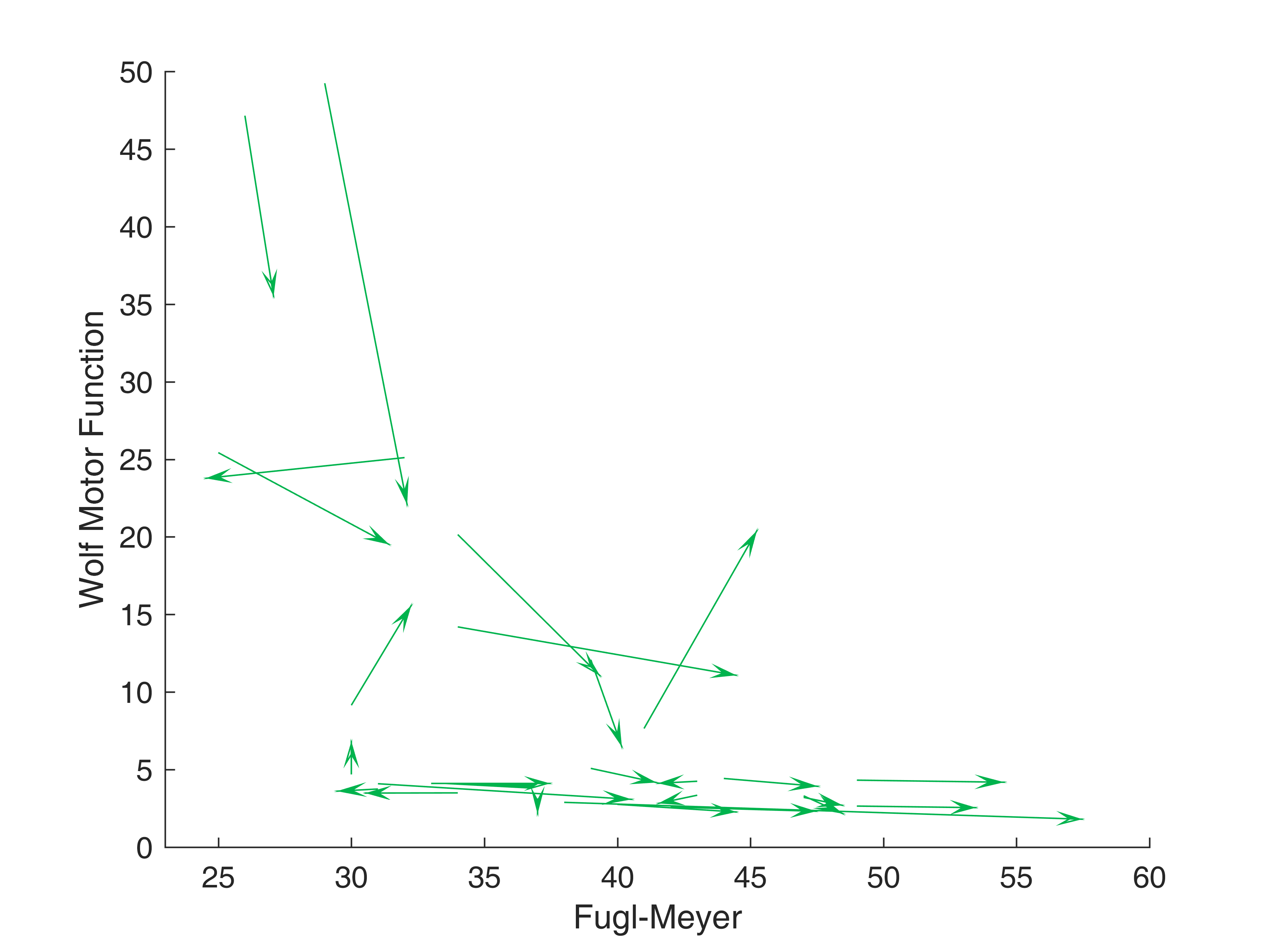

Supplement: S6 Fig — UEFM was more sensitive to patients with relatively higher functional ability, while WMFT was more sensitive to those with lower functional ability. WMFT scores plateaued for patients showing larger UEFM changes. This relationship between UEFM and WMFT may explain our observation that slower speed predicted better recovery for UEFM while higher speeds were predictive of faster WMFT times. Changes in clinical scores were not statistically significant after the intervention. (TIF) [file pone.0205639.s006.tif]
